# A case against the trickle-down effect in technology ecosystems

**DOI:** 10.1371/journal.pone.0218370

**Published:** 2019-06-13

**Authors:** Guanglu Zhang, Douglas Allaire, Venkatesh Shankar, Daniel A. McAdams

**Affiliations:** 1 Department of Mechanical Engineering, Texas A&M University, College Station, TX, United States of America; 2 Department of Marketing and Center for Retailing Studies, Mays Business School, Texas A&M University, College Station, TX, United States of America; Pavol Jozef Safarik University in Kosice, SLOVAKIA

## Abstract

Technology evolution describes a change in a technology performance over time. The modeling of technology evolution is crucial for designers, entrepreneurs, and government officials to set reasonable R&D targets, invest in promising technology, and develop effective incentive policies. Scientists and engineers have developed several mathematical functions such as logistic function and exponential function (Moore’s Law) to model technology evolution. However, these models focus on how a technology evolves in isolation and do not consider how the technology interacts with other technologies. Here, we extend the Lotka-Volterra equations from community ecology to model a technology ecosystem with system, component, and fundamental layers. We model the technology ecosystem of passenger aircraft using the Lotka-Volterra equations. The results show limited trickle-down effect in the technology ecosystem, where we refer to the impact from an upper layer technology to a lower layer technology as a trickle-down effect. The limited trickle-down effect suggests that the advance of the system technology (passenger aircraft) is not able to automatically promote the performance of the component technology (turbofan aero-engine) and the fundamental technology (engine blade superalloy) that constitute the system. Our research warns that it may not be effective to maintain the prosperity of a technology ecosystem through government incentives on system technologies only. Decision makers should consider supporting the innovations of key component or fundamental technologies.

## Introduction

Technology evolution describes a change in a technology performance over time. The modeling and fundamental understanding of technology evolution is crucial for designers, entrepreneurs, and government officials. Among other uses, such understanding informs decision makers as they set reasonable R&D targets, invest in promising technology, and develop effective incentive policies [1–3]. Scientists and engineers have developed mathematical functions to model technology evolution [4–6]. For example, Moore’s Law is an exponential function that is widely used in the semiconductor industry to model the change in microprocessor transistor count over time [7]. These mathematical functions, such as Moore’s Law and logistic function, model a single technology’s evolution in isolation from those of related technologies. However, many technologies are part of a technology ecosystem [8, 9], comprising system technology, component technology, and fundamental technology layers. For example, a smartphone, a microprocessor, and lithographic technology represent technologies that live in the smartphone technology ecosystem’s system, component, and fundamental layers, respectively. Existing technology evolution models do not generally consider the interaction among the technologies in a technology ecosystem.

We extend the Lotka-Volterra equations from community ecology [10] to model the technology ecosystem rather than a single technology as is typically done in literature. The Lotka-Volterra ecosystem model captures the interaction between technologies, enabling us to quantify any trickle-down effect in technology ecosystems. A trickle-down effect refers to the impact from an upper layer technology to a lower layer technology in a technology ecosystem, consistent with a similar definition in economics [11]. Government officials potentially assume that a significant trickle-down effect exists in any technology ecosystem when they develop technology policies and incentives. Many may believe that the advance of a system technology can automatically enhance the performances of its component and fundamental technologies, and that beneficial resources such as R&D capital naturally flow down to the lower layers in a technology ecosystem. Based on this belief, government incentives are often designed for only system technologies and not for the technologies that live in the lower layers of the ecosystem. For example, several national governments have provided substantial tax exemptions or tax credits for electric automobiles (system technology) [12]; until 2016, the US government has provided more than $176 billion subsidies to the biggest wind-turbine manufacturers (system technology developers) [13]. Although a significant trickle-down effect is an underlying assumption behind many government incentives, there exists no rigorous research approach that quantifies the trickle-down effect in a technology ecosystem.

In this paper, we propose an approach to quantify the trickle-down effect by studying the interaction between system, component, and fundamental technologies through an application of the Lotka-Volterra ecosystem model in a case study of passenger aircraft. To our knowledge, the successful application of the Lotka-Volterra ecosystem model to quantify trickle-down effect in a technology ecosystem is a novel contribution to technology evolution modeling. The results of our research show limited trickle-down effect in the technology ecosystem of passenger aircraft. The limited trickle-down effect suggests that it may not be effective to invigorate a technology ecosystem through investments in, or subsidies for, only system technologies. More resources should be allocated toward the innovation of key component or fundamental technologies.

## Lotka-Volterra ecosystem model

Vito Volterra introduced the Lotka-Volterra equations in the early 20th century to model the population changes of sharks and fishes in the Adriatic Sea. The mathematical form of the Lotka-Volterra equations have been modified and successfully applied in community ecology and demography during the last century [14, 15].

Pistorius and Utterback suggested studying the interaction between two technologies using generalized Lotka–Volterra equations in 1997 [16]. Modis also employed the Lotka-Volterra equations to model product sales in 1997 [17]. Lotka-Volterra equations then have been applied in a variety of contexts following these pioneering works. Lee et al. analyzed two Korean stock markets in using Lotka-Volterra equations in 2005 [18]. Marasco et al. used Lotka-Volterra equations to study market share dynamics between two or more competitors in 2016 [19]. Zhang et al. extended the generalized Lotka-Volterra equations to model the interactions between one system and one or more components in 2018 and 2019 [20, 21].

**Fig 1:**
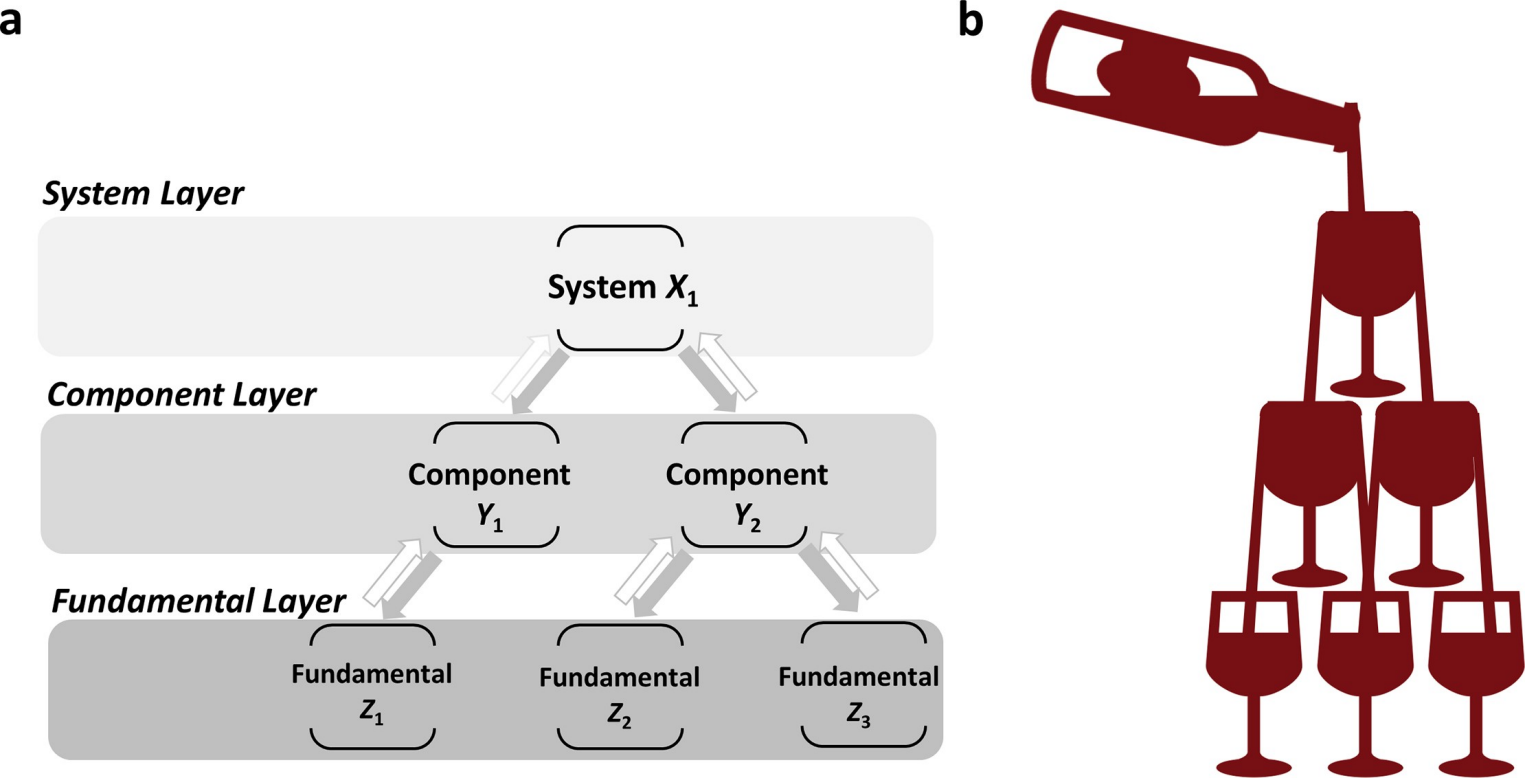
Hierarchical technology ecosystem and trickle-down effect in technology evolution. a, A typical technology ecosystem includes three layers, system layer, component layer, and fundamental layer. The grey arrows represent the trickle-down effect, while the white arrows denote the trickle-up effect in technology evolution. b, If trickle-down effect is significant in technology evolution, the improvement of system technology will drive and grow the performance of component and fundamental technologies.

In this paper, we focus on modeling the technical performance (e.g., speed, capacity, and energy efficiency) of technologies rather than the business indicators of technologies, such as cost, price, production, sales revenue, and profit [6, 22, 23]. We note that an engineering system lives in a technology ecosystem that includes at least three layers. Prior research has modeled only the interaction between a system and its components (i.e., two layer interaction). Fig 1A shows a typical hierarchical technology ecosystem that includes a system layer, a component layer, and a fundamental layer. The system layer contains the system technology that meets one or more functional requirements for end users or stakeholders. Given this abstraction, the system technology is realized through the integration of multiple hardware and software elements, commonly referred to as component technologies. Component technologies support the system technology but usually cannot fulfill users’ functional requirements separately. These component technologies establish a component layer in the technology ecosystem. Furthermore, fundamental technologies enable the invention of component technologies. Each component technology consists of several fundamental technologies that constitute the fundamental layer in the technology ecosystem. For example, a microprocessor is a key component of a smartphone system. Improvements in microprocessor performance enable enhanced smartphone performance. In turn, this performance enhancement of the microprocessor relies on the advancement of lithographic technology. If significant trickle-down effect exists in a technology ecosystem, the performance improvement in the system technology will drive and grow the performance of the component and fundamental technologies (Fig 1B). In this case, technical investment is only needed at the system level. It is not necessary to directly invest in the technologies that belong to the component or fundamental layer.

We use a Lotka-Volterra equation to quantitatively model the technical performance evolution of each technology and quantify the trickle-down effect in technology ecosystems that include at least three layers. A set of coupled ordinary differential equations describe the whole ecosystem. The Lotka-Volterra ecosystem model for the hierarchical technology ecosystem shown in Fig 1A includes six differential equations. For example, the equation for component technology *Y*_1_ is

$$\frac{dy_{1}}{dt}=A_{y_{1}}y_{1}-B_{y_{1}}y_{1}^{2}+C_{y_{1}x_{1}}y_{1}x_{1}+C_{y_{1}z_{1}}y_{1}z_{1},$$ (1)

where *y*_1_ is the performance of component technology *Y*_1_, *x*_1_ is the performance of system technology *X*_1_, *z*_1_ is the performance of fundamental technology *Z*_1_; *y*_1_, *x*_1_, and *z*_1_ are functions of time *t*; *A*, *B*, and *C* denote estimated parameters derived from a model fitting process.

The parameters *A*, *B*, and *C* in the Lotka-Volterra ecosystem model are associated with causal factors of technology evolution [20]. Parameter *A* represents the independent growth rate of technology performance. The value of parameter *A* depends on R&D investment, government incentives, and other stimulation factors. Parameter *B* indicates technical difficulty. Technical difficulty is the challenge that a technology has to overcome for performance improvement. The value of parameter *B* also sets the upper limit value of technology performance. Parameter *C* describes the interaction between technologies and reflects the ecological dependency. A larger value of parameter *C* indicates a greater dependency on one technology than another. For example, if the component technology *Y*_1_ has a greater impact on the system technology *X*_1_ than that of the component technology *Y*_2_, the parameter *Cx*_1_*y*_1_ will have a larger value than the parameter *Cx*_1_*y*_2_.

The trickle-down effect could be verified through the value of parameter *C* in the Lotka-Volterra ecosystem model. If each parameter *C* that represents the trickle-down effect is great larger than the other parameter *C* (e.g., *Cy*_1_*x*_1_>> *Cy*_1_*z*_1_ in Eq 1), we can conclude that a significant trickle-down effect exists in the technology ecosystem.

## Results and discussion

**Fig 2:**
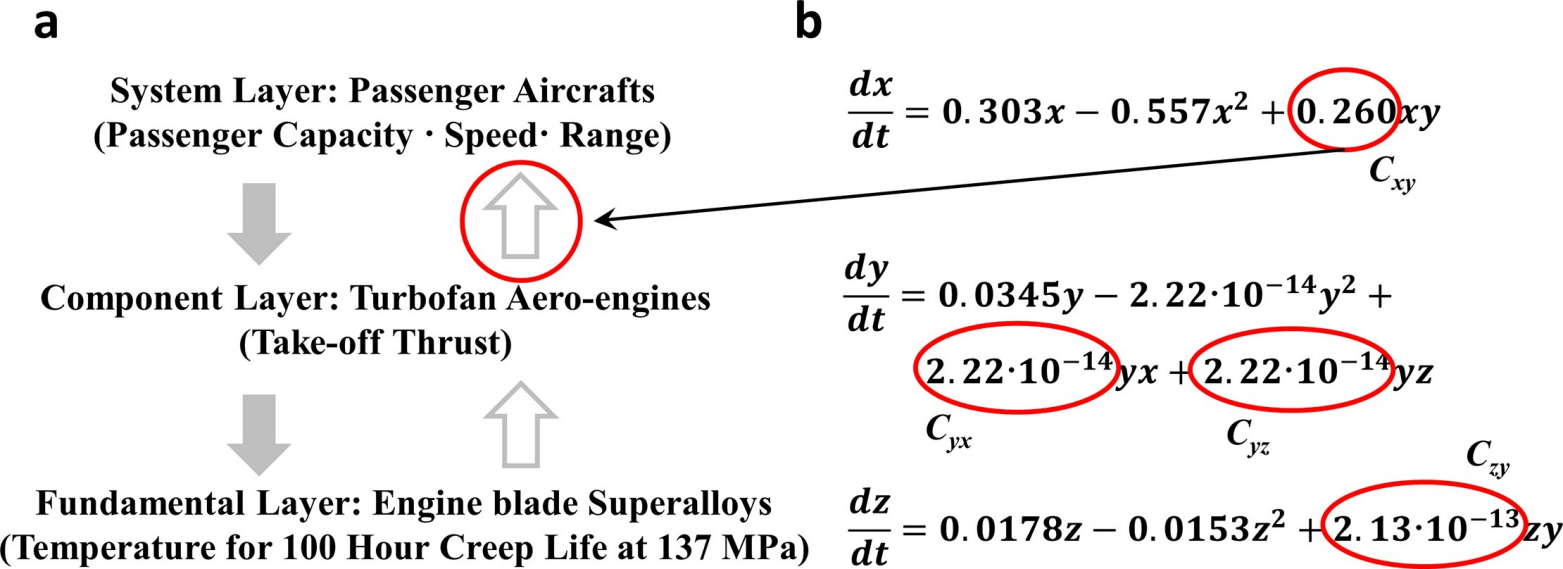
A simplified Lotka-Volterra ecosystem model for passenger aircraft ecosystem. a, We include only one technology in each layer of the technology ecosystem and denote the trickle-down effect by grey arrows. b, The model fitting results indicate limited trickle-down effect in the passenger aircraft ecosystem. Among the four technology interactions denoted by the grey and white arrows in the figure, only the impact from the component technology (turbofan aero-engine) on the system technology (passenger aircraft) is significant.

We build a simplified hierarchical ecosystem for passenger aircraft as shown in Fig 2A to test the trickle-down effect and understand other technology interactions in the ecosystem. For this study, we only include one technology in each layer of the technology ecosystem. Here, passenger aircraft is the system technology, turbofan aero-engine is the component technology, and engine blade superalloy is the fundamental technology. The grey and white arrows in Fig 2A denote the interactions among these three technologies in the ecosystem. In this ecosystem, the Lotka-Volterra ecosystem model has three equations. We choose a major performance metric for each technology and collect historical data from 1960 to 2010. We fit the Lotka-Volterra ecosystem model to the data and estimate the values of parameters *A*, *B*, and *C* in the model using least squares data fitting [24]. The best estimated parameter values derived through least squares data fitting minimize the sum of squared residuals, where a residual is the difference between an observed technology performance and the fitted technology performance provided by the Lotka-Volterra ecosystem model. Fig 2B shows the model fitting results. The parameter *C* in each equation corresponds to an arrow in Fig 2A. The value of parameter *C* represents the extent of impact from one technology on the other technology. We find a significant impact only from the turbofan aero-engine (component technology) on the passenger aircraft (system technology), represented by the 0.256*xy* term in the Lotka-Volterra equation for the system technology. The values of parameter *C* in the other two equations are small enough to be zero (i.e., 2.22⋅10^−14^, 2.22⋅10^−14^, and 2.13⋅10^−13^). These small values indicate limited technology interactions. We interpret these results to reveal a limited trickle-down effect, an isolated component technology, and a fundamental technology barrier in the passenger aircraft technology ecosystem.

The parameters *Cyx* and *Czy* represent the trickle-down effect in the technology ecosystem of passenger aircraft. The values of these two parameters are 2.22⋅10^−14^ and 2.13⋅10^−13^ respectively. These results show that the impact from the upper layer technology on the lower layer technology is limited in the technology ecosystem. Although the development of an upper layer technology usually demands that its supportive technologies have better performance, which could promote a synergistic relationship between the technology developers or manufacturers, the model results in Fig 2B show a *limited trickle-down effect* in the passenger aircraft ecosystem. The limited trickle-down effect may result from limited beneficial resources such as R&D capital that naturally flow down to the lower layers in the technology ecosystem. Thus, if a decision making agency such as the government wants to invigorate the entire technology ecosystem of passenger aircrafts, it may be not effective to invest only in the system technology developers or manufacturers. The decision maker should also consider allocating resources to the development of component and fundamental technologies.

Fig 2B also shows that both the passenger aircraft (system technology) and the engine blade superalloy (fundamental technology) have limited effect on the turbofan aero-engine (component technology) as represented by the small values of parameters *Cyx* and *Cyz*. The evolution of the turbofan aero-engine follows Moore’s Law since a Lotka-Volterra equation reduces to Moore’s Law when parameters *B* and *C* in Eq (1) equal zero [15, 25]. The *isolated component technology* needs more research and development (R&D) attention because it has a significant impact on the system technology but cannot leverage both the system technology and the fundamental technology evolution in the case of the passenger aircraft ecosystem. Investment in the R&D of the turbofan aero-engine is the only effective way to improve its performance. Such investment can bring high returns from the ecosystem perspective because it indirectly improves the performance of passenger aircraft (system technology).

The model results for the passenger aircraft ecosystem also indicate limited impact from the engine blade superalloy (fundamental technology) on the turbofan aero-engines (component technology) through the small value of parameter *Cyz*. Such a result is counterintuitive because scientists typically emphasize the importance of the fundamental technology in a technology ecosystem. In fact, our result confirms that the fundamental technology is important at the beginning of the component technology evolution. We observe a *fundamental technology barrier* in the evolution of passenger aircraft. When the turbofan aero-engine substituted for the shaft aero-engine in 1960s, the performance of engine blade superalloy had reached 87% of its performance in 2005 [26]. We hypothesize from this observation that the fundamental technology is critical for the new component technology invention but has limited impact on the subsequent incremental changes in the new component technology.

We caution against generalizing these results to other technology ecosystems because these results are based on a simplified hierarchical ecosystem of passenger aircraft. Moreover, we assume the parameters in the Lotka-Volterra ecosystem model as constants, but the causal factors associated with the parameters may vary over time in reality, changing the parameters from constants to functions of time. To generalize our findings, future research should study other technology ecosystems in diverse industries and model the parameters as functions of time if necessary [19, 27]. Despite these limitations, the modeling results of the passenger aircraft ecosystem do provide important pointers to designers, entrepreneurs, and government officials when they make important decisions on technology development. Specifically, it is unreasonable to assume that a significant trickle-down effect exists in any technology ecosystem. The advance of an upper layer technology may not automatically enhance the performance of lower layer technologies. Our research warns that it may not be effective to maintain the prosperity of a technology ecosystem through R&D investment or government incentives on system technologies. Beneficial resources such as R&D capital may not naturally flow down to the lower layers in the technology ecosystem. Instead, based on the significant impact from the component technology on the system technology in our passenger aircraft case study, decision makers should consider investing in key component technologies to promote the incremental change of system technology or funding research on promising fundamental technologies to facilitate component technology substitution in the technology ecosystem.

## Supporting information

S1 File Supplementary materials. (DOCX)
