# Supplementary material for: A case against the trickle-down effect in technology ecosystems
Source: PLoS One. 2019 Jun 13;14(6):e0218370. doi: 10.1371/journal.pone.0218370 (PMC6564689; doi:10.1371/journal.pone.0218370)
Supplement: S1 File — (DOCX) [file pone.0218370.s001.docx]

A case against the trickle-down effect in technology ecosystems

Supplementary materials

Guanglu Zhang, Douglas Allaire, Venkatesh Shankar, Daniel A. McAdams

May 20, 2019

**Data collection and pretreatment of passenger aircraft technology ecosystem.** We use a combined metric of passenger capacity⬝speed⬝range (km^2^/h) to describe the change in passenger aircraft performance. The three major independent component technologies that support passenger aircraft are aerodynamics, structures, and propulsion [1]. These three component technologies have the same level of impact on passenger aircraft performance [1]. We include propulsion (turbofan aero-engine) as the only component technology in this case study and use take-off thrust (kN) as its performance metric. We select engine blade superalloy as the fundamental technology that supports turbofan aero-engine development [2]. We use temperature (°C) for 100 hour creep life at 137 MPa as the superalloy performance metric.

We believe that this simplification is acceptable because aerodynamics and structures of passenger aircraft have limited improvements compared with significant advance in propulsion during 1960-2010. Specifically, the wing aspect ratio and payload divided by typical airline operating empty weight (OEW), which are the performance metrics of aerodynamics and structure of passenger aircraft, displayed improvements of 48% and 36%, respectively, during 1960-2010. During the same time period, take-off thrust of the turbofan aero-engine showed a 653% improvement. Furthermore, neither do we consider aerodynamics nor structures as component technologies in this case study because the data of their fundamental technologies is not readily available. Of note, we realize that the similar simplification (i.e., include only one major component technology in a technology ecosystem) may not be valid to model any other technology ecosystems.

We compile the passenger aircraft performance data (e.g., maximum passenger capacity, first flight year, wing aspect ratio, payload, and OEM) from multiple volumes of Jane's All the World's Aircraft and WIKIPEDIA. We collect the turbofan aero-engine data from the ICAO databank [3] and digitize engine blade superalloy data from a figure in the book written by Reed [4].

We collect the performance data of each technology from 1960 to 2010 and select the peak performance data every year to include in our modeling. We also remove a data point if its performance value is smaller than that of any previous data points because each data point should represent the best available technology performance during the time period. For example, there are 98 passenger aircraft data points measured by passenger capacity⬝speed⬝range (km^2^/h) during 1960-2010. Among these 98 data points, we select only 10 data points (shown as the points in Figure 1) to represent the evolution of passenger aircraft performance from 1960 to 2010.

To normalize the data for comparison, we non-dimensionalize the selected data sets before subsequent model fitting. We divide each data point by its corresponding maximum performance value during modeling time interval. The value of every data point is within the range (0, 1] after the non-dimensionalized treatment.

**Lotka-Volterra ecosystem model fitting of passenger aircraft technology ecosystem.** We build the Lotka-Volterra ecosystem model based on the simplified hierarchical ecosystem in main text as

|  | $\frac{dx}{dt}=A_{x}x-B_{x}x^{2}+C_{xy}xy$ | (1) |
| --- | --- | --- |
|  | $\frac{dy}{dt}=A_{y}y-B_{y}y^{2}+C_{yx}yx+C_{yz}yz$ | (2) |
|  | $\frac{dz}{dt}=A_{z}z-B_{z}z^{2}+C_{zy}zy$ | (3) |

where *x* is the non-dimensionalized passenger aircraft performance (passenger capacity⬝speed⬝range), *y* is the non-dimensionalized take-off thrust of turbofan aero-engine, *z* is the non-dimensionalized engine blade superalloy performance (temperature for 100 hour creep life at 137 MPa), *A_x_*, *A_y_*, *A_z_*, *B_x_*, *B_y_*, *B_z_*, *C_xy_*, *C_yx_*, *C_yz_*, and *C_zy_* are constant parameters estimated from model fitting. Here, we assume that the system technology does not have direct interaction with the fundamental technology, so Eq. (1) and Eq. (3) have only one *C* term each.

The range of each parameter in Eqs. (1) - (3) is (0, +∞). We also set the initial values of each equation as unknown parameters with the range (0, 1). In total, there are 13 unknown parameters in the Lotka-Volterra ecosystem model. We use the trust region reflective algorithm [5] to search the parameter space (range). In each search step, we employ the high order Runge-Kutta method [6, 7] to solve Eqs. (1) - (3) numerically. We take multi-start and derive the values of the 13 parameters that minimize the sum of squared errors between the technology performance data sets and the solutions of Eqs. (1) - (3). We plug the parameter values into Eqs. (1) - (3) to obtain the following equations

|  | $\frac{dx}{dt}=0.303x-0.557x^{2}+0.260xy$ | (4) |
| --- | --- | --- |
|  | $\frac{dy}{dt}=0.0345y-2.22\cdot{10}^{-14}y^{2}+2.22\cdot{10}^{-14}yx+2.22\cdot{10}^{-14}yz$ | (5) |
|  | $\frac{dz}{dt}=0.0178z-0.0153z^{2}+2.13\cdot{10}^{-13}zy$ | (6) |
|  | $x(t=0)=$ 0.07169 | (7) |
|  | $y(t=0)=0.2245$ | (8) |
|  | $z(t=0)=0.8540$ | (9) |

where *t*=0 represents the year 1960. The model fitting results of system, component, and fundamental technologies appear in Figures 1, 2, and 3, respectively. We show Eqs. (4) - (6) in main text.


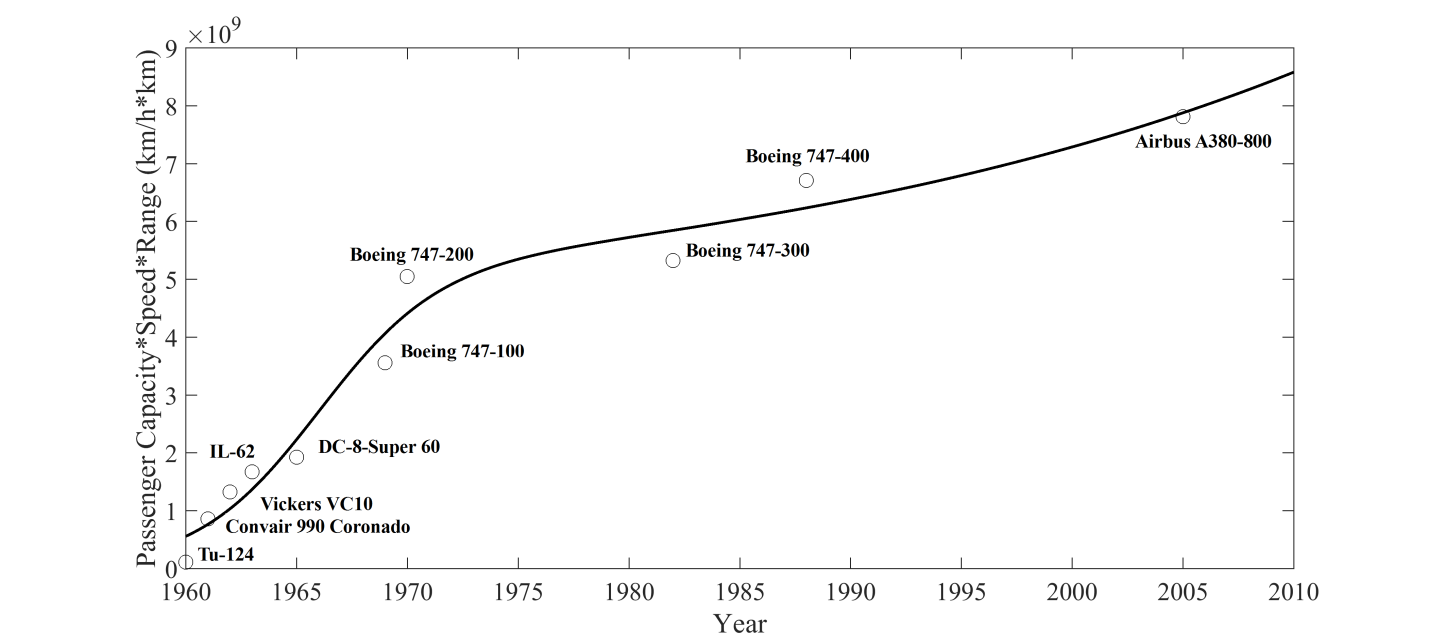


Figure 1. Lotka-Volterra ecosystem modeling result of passenger aircraft (system technology) performance evolution during 1960-2010. The performance metric is passenger capacity⬝speed⬝range (km^2^/h).


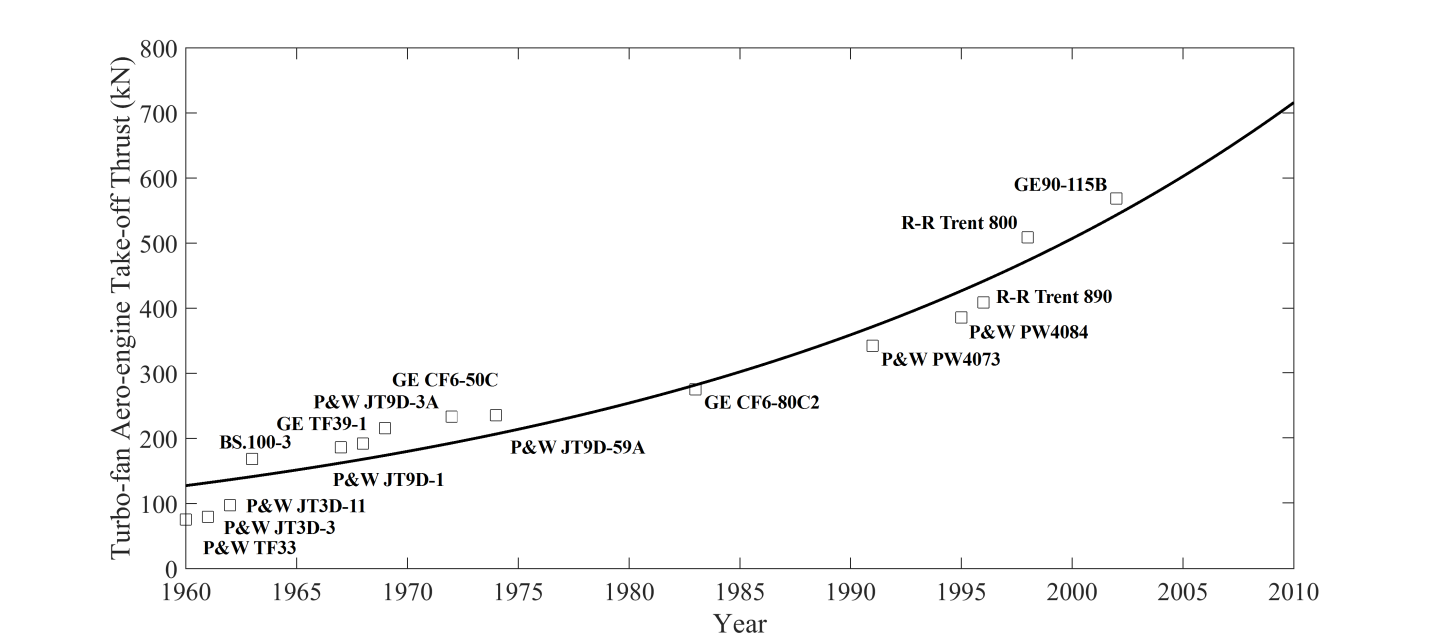


**Figure 2. Lotka-Volterra ecosystem modeling result of turbofan aero-engine (component technology) performance evolution during 1960-2010.** The performance metric is take-off thrust (kN).


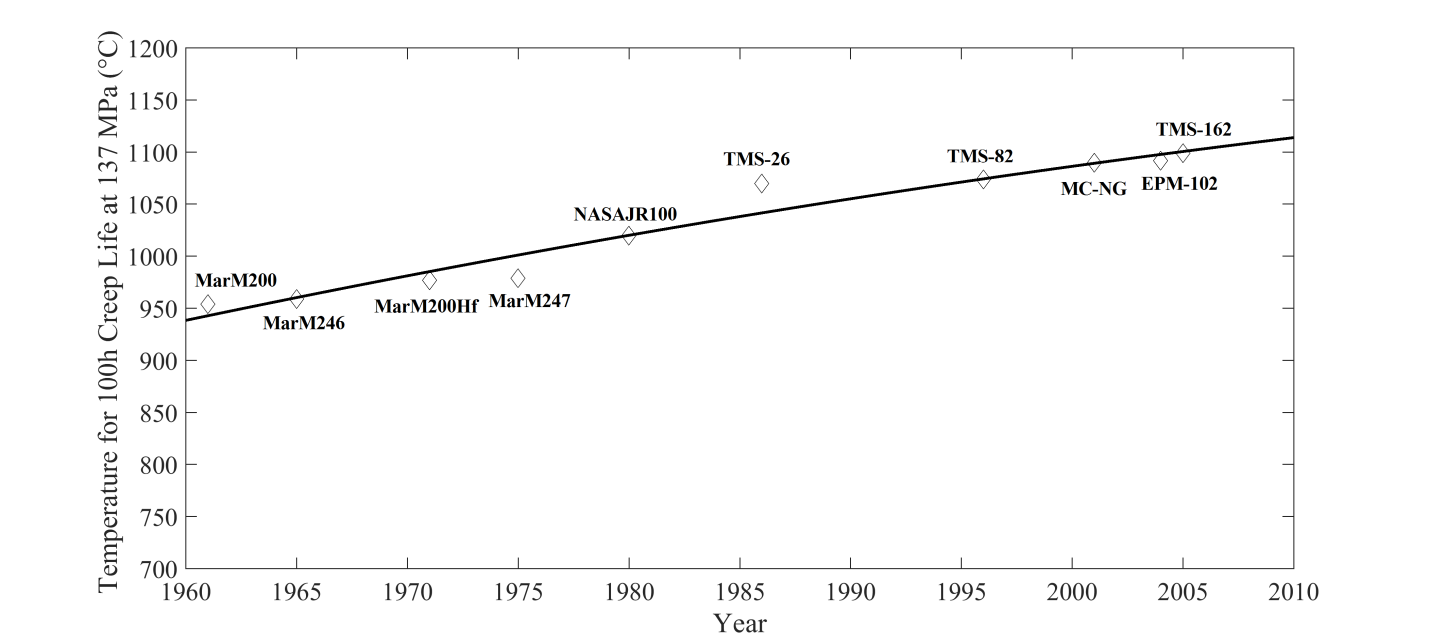


**Figure 3. Lotka-Volterra ecosystem modeling result of engine blade superalloy (fundamental technology) performance evolution during 1960-2010.** The performance metric is temperature (°C) for 100 hour creep life at 137 MPa.

Table 1. Passenger aircraft (system technology) performance evolution data during 1960-2010.

| Year | Name | Passenger Capacity | Speed (km/h) | Range (km) | Capacity*Speed*Range |
| --- | --- | --- | --- | --- | --- |
| 1960 | Tupolev Tu-124 | 56 | 970 | 2100 | 114072000 |
| 1961 | Convair 990 Coronado | 149 | 1000 | 5785 | 861965000 |
| 1962 | Vickers VC10 | 151 | 933 | 9412 | 1325990796 |
| 1963 | Ilyushin IL-62 | 186 | 900 | 10000 | 1674000000 |
| 1965 | Douglas DC-8-Super 60 Series | 259 | 895 | 8334 | 1931862870 |
| 1969 | Boeing 747-100 | 366 | 1136 | 8560 | 3559042560 |
| 1970 | Boeing 747-200 | 366 | 1136 | 12150 | 5051678400 |
| 1982 | Boeing 747-300 | 400 | 1136 | 11720 | 5325568000 |
| 1988 | Boeing 747-400 | 416 | 1136 | 14200 | 6710579200 |
| 2005 | Airbus A380-800 | 544 | 945 | 15200 | 7814016000 |

**Table 2. Turbofan aero-engine (component technology) performance evolution data during 1960-2010.**

| Year | Name | Take-off Thrust (lb) | Take-off Thrust (kN) |
| --- | --- | --- | --- |
| 1960 | Pratt & Whitney TF33 (JT3D-1) | 17000 | 75.62 |
| 1961 | Pratt & Whitney JT3D-3 | 18000 | 80.07 |
| 1962 | Pratt & Whitney JT3D-11 | 22000 | 97.86 |
| 1963 | Bristol Siddeley BS.100-3 | 38000 | 169.03 |
| 1967 | Pratt & Whitney JT9D-1 | 42000 | 186.83 |
| 1968 | General Electric TF39-1 | 43300 | 192.61 |
| 1969 | Pratt & Whitney JT9D-3A | 48500 | 215.74 |
| 1972 | General Electric CF6-50C | 52500 | 233.53 |
| 1974 | Pratt & Whitney JT9D-59A | 53000 | 235.76 |
| 1983 | General Electric CF6-80C2 | 62000 | 275.79 |
| 1991 | Pratt & Whitney PW4073 | 77000 | 342.51 |
| 1995 | Pratt & Whitney PW4084 | 86760 | 385.93 |
| 1996 | Rolls-Royce Trent 890 | 92000 | 409.24 |
| 1998 | Rolls-Royce Trent 800 | 114500 | 509.32 |
| 2002 | General Electric GE90-115B | 127900 | 568.93 |

**Table 3. Engine blade superalloy (fundamental technology) performance evolution data during 1960-2010.**

| Type | Name | Year | Temperature (°C) |
| --- | --- | --- | --- |
| Conventionally Cast | MarM200 | 1961 | 954 |
| Conventionally Cast | MarM246 | 1965 | 959 |
| Directionally Solidified | MarM200Hf | 1971 | 977 |
| Directionally Solidified | MarM247 | 1975 | 979 |
| Single Crystal | NASAJR100 | 1980 | 1020 |
| Single Crystal | TMS-26 | 1986 | 1070 |
| Single Crystal | TMS-82 | 1996 | 1074 |
| Single Crystal | MC-NG | 2001 | 1090 |
| Single Crystal | EPM-102 | 2004 | 1092 |
| Single Crystal | TMS-162 | 2005 | 1099 |

**References**

1. Raymer D. Aircraft design: A conceptual approach. Reston, Virginia, USA: American Institute of Aeronautics and Astronautics, Inc.; 2012.

2. Schafrik R, Sprague R. Superalloy technology - a perspective on critical innovations for turbine engines. Key Engineering Materials. 2008;380:113-34.

3. EASA. ICAO Aircraft engine emissions databank. European Aviation Safety Agency, 2016.

4. Reed RC. The superalloys: Fundamentals and applications. Cambridge, UK: Cambridge University Press; 2006.

5. Moré J, Sorensen D. Computing a trust region step. SIAM Journal on Scientific and Statistical Computing. 1983;4(3):553-72.

6. Lapidus L, Seinfeld JH. Numerical solution of ordinary differential equations. New York: Academic Press; 1971.

7. Dormand JR, Prince PJ. A family of embedded Runge-Kutta formulae. Journal of Computational and Applied Mathematics. 1980;6(1):19-26.
